# Template‐Assisted 3D‐PVDF Nanonetworks for Passive Daytime Radiative Cooling

**DOI:** 10.1002/nap2.70183

**Published:** 2026-06-29

**Authors:** Amaia Iglesias‐Elcano, Luis Moreno‐Sanabria, Marisol Martín‐González, Cristina V. Manzano

**Affiliations:** ^1^ Instituto de Micro y Nanotecnología, IMN‐CNM, CSIC (CEI UAM+CSIC) Tres Cantos Madrid Spain

**Keywords:** anodic aluminum oxide, optical properties, polyvinylidene fluoride, radiative coolers, UV treatment

## Abstract

Passive daytime radiative cooling (PDRC), which dissipates heat through infrared emission to outer space while reflecting solar radiation, represents a promising strategy for reducing energy consumption in cooling technologies. Here, a template‐assisted strategy is introduced to fabricate nanostructured polyvinylidene fluoride (PVDF) radiative coolers via polymer infiltration into three‐dimensional anodic aluminum oxide (3D‐AAO) nanoporous templates. The optical properties and cooling performance of the 3D‐PVDF nanostructures are investigated with and without the AAO template. To tune the crystalline phase composition and its influence on optical response, three post‐infiltration cooling methods—natural, fast, and ultra‐fast—are applied. A UV treatment further enhances solar reflectance through photo‐oxidation‐induced whitening. The optimized free‐standing 3D‐PVDF obtained via ultra‐fast cooling exhibits an average solar reflectance of 82.4% (0.32–2.5 μm) and an infrared emissivity of 96.7% within the 8–13 μm atmospheric window. These properties correspond to a theoretical daytime cooling power density of 182.3 W m^−2^ under a solar irradiance of 1000 W m^−2^. Outdoor measurements demonstrate a daytime temperature reduction of 12.9°C under peak solar irradiance of 962 W m^−2^ relative to an empty reference box. This work establishes a scalable strategy for engineering three‐dimensional polymer nanostructures for high‐performance radiative cooling and energy‐efficient building technologies.

## Graphical abstract

Three‐dimensional polyvinylidene fluoride (3D‐PVDF) nanostructures were fabricated by infiltrating the polymer into 3D anodic aluminum oxide (3D‐AAO) templates. Due to their high solar reflectance (82.4%) and infrared emissivity (96.7% between 8 and 13 µm), they are an ideal candidate for radiative cooling. These 3D nanostructures achieve a cooling temperature of 12.9°C under 960 W m^−2^ with a calculated cooling power of 182 W m^−2^.

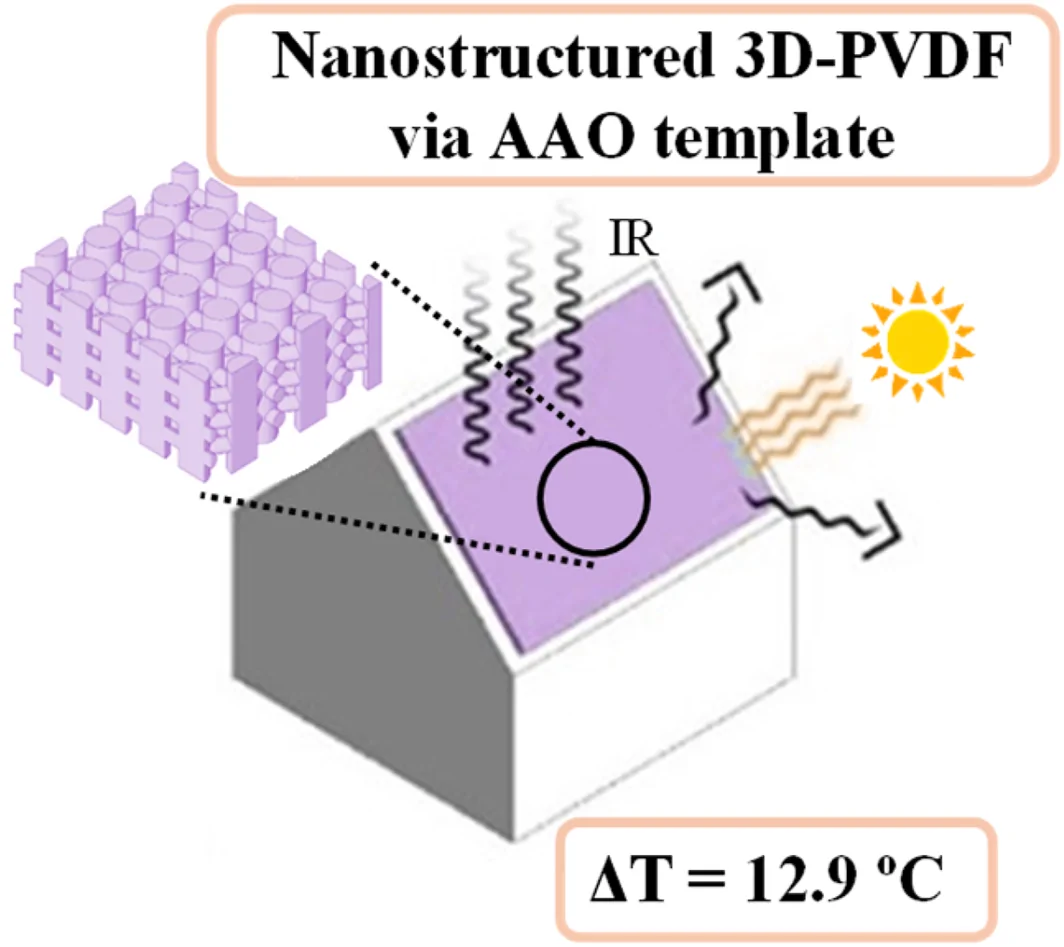

## 1 Introduction

Over the past decades, global energy consumption associated with refrigeration has increased substantially, currently accounting for approximately 20% of total electricity use worldwide. This demand is expected to grow in the following decades, rising from about 2000 TWh in 2020 to more than 6000 TWh in 2050 [1]. In our current context, characterized by climate change, rising mean temperatures, and environmental pollution, sustainable alternatives to conventional cooling methods are necessary. Most of these conventional systems rely heavily on nonrenewable energy sources and contribute to environmental heating by releasing waste heat into the immediate surroundings [2]. Among the emerging solutions, passive daytime radiative cooling (PDRC) is particularly promising. The underlying physical principle of PDRC relies on the material's ability to emit thermal energy as infrared radiation toward outer space, acting as a remote heat sink, by exploiting the atmospheric transparency window (8–13 μm). The material must also exhibit high reflectance toward incident solar radiation (0.3–2.5 μm) [3]. Thus, under favorable atmospheric and thermal conditions, this combination can lead to a net negative heat balance, enabling the material to reach sub‐ambient temperatures passively, without the need for an external power supply.

Among the materials explored for this application, polymers exhibit great potential due to their high mid‐infrared emissivity, arising from the vibrational modes of their functional groups, particularly within the 8–13 μm atmospheric window, where characteristic C–H, C–O, C–Cl, C–F, and C–N bond vibrations occur [4]. Additionally, polymers generally present low extinction coefficients across the solar spectral range, leading to reduced solar absorption [5]. Since reflectance (*R*), absorbance (*A*), and transmittance (*T*) are related through *R* = 1 − *A* − *T*, the solar reflectance (*R*_solar_) of polymers is strongly governed by their intrinsic transparency to solar radiation [6]. To further enhance *R*_solar_, different approaches have been reported in the literature, including the use of metallic reflective layers, the incorporation of dielectric nanoparticles, and micro‐ or nanostructuring of the polymer to increase light scattering [5, 7, 8]. Some of the most extensively investigated polymers for this application include cellulose acetate (CA), polymethyl methacrylate (PMMA), polydimethylsiloxane (PDMS), and polyvinylidene fluoride (PVDF) [9, 10, 11, 12].

As previously mentioned, PVDF has been extensively investigated over the past decade for radiative cooling applications. PVDF is one of the most important polymers for radiative cooling thanks to its unique combination of strong C–F and C–H polar bonds, which result in strong emission between 8 and 13 μm [13]. It is extremely UV‐resistant and has excellent outdoor weatherability and durability [14]. PVDF is commonly known as a hydrophobic polymer, making it effectively self‐cleaning [15]. Its mechanical flexibility also makes it ideal for potential applications in buildings, roofs, vehicles, electronics, and textiles [16].

In most studies, it is combined with other materials, ranging from polymer blends with hexafluoropropylene (HFP), polymethyl methacrylate (PMMA) or polydimethylsiloxane (PDMS) to composites containing TiO_2_, SiO_2_ or Al_2_O_3_ nanoparticles, or combinations thereof [17, 18, 19, 20, 21, 22, 23]. A key milestone was reported by Mandal et al., who fabricated hierarchically porous PVDF‐HFP, achieving a sub‐ambient temperature reduction of 6°C and a cooling power density of 96 W m^−2^ under solar irradiances of 890 and 750 W m^−2^, respectively, thereby demonstrating the potential of PVDF as an effective radiative cooling material [24]. Following this work, a wide range of PVDF‐based coolers have been developed. Recent examples include a hierarchical micro‐ and nanoporous PVDF aerogel with a cooling of 12.1°C under 1200 W m^−2^ solar radiation, and an anisotropic PVDF‐HFP porous coating with a daytime temperature drop of ∼ 6.3°C [25, 26]. The incorporation of alumina (Al_2_O_3_) as nanoparticles within PVDF fibers or as microspheres within PVDF‐HFP composites enabled sub‐ambient cooling temperatures of 4.0°C and 9.1°C, with associated cooling power densities of 82.7 W m^−2^ and 87.2 W m^−2^, respectively [8, 27]. These materials are predominantly fabricated using phase inversion techniques, although alternative approaches such as electrospinning and templating have also been explored [28]. In the latter, Huang et al. employed MOF sacrificial templates to produce hierarchical nanoporous PVDF with pores measuring approximately 400 nm, achieving sub‐ambient cooling of ∼ 9.5°C and a cooling power density of 137 W m^−2^ [18]. Important findings have also been reported by Chen et al., where a validated model demonstrates the weight of different lattice parameters (pore size, porosity and thickness) in the optical properties of a PVDF‐HFP copolymer [29] and a novel “bottom‐up” ball milling approach to create uniform micro‐assembly of P(VDF‐HFP) nanoparticles with high radiative cooling performance was reported [30]. These studies collectively reinforce the importance of controlling polymer architecture at the nanoscale to optimize optical performance in PVDF‐based radiative coolers.

Precise control over the geometry of the material during fabrication is crucial, since the optical properties of radiative coolers are strongly influenced by their structural design. Following a template‐assisted approach, this work proposes the use of three‐dimensional anodic aluminum oxide (3D‐AAO, also known as NAA, NAAP, or PAA) scalable nanoporous templates to fabricate nanostructured PVDF. The fabrication of 3D‐AAO templates has been widely explored, with applications including photonic structures, template‐assisted nanostructure fabrication, RGB color technologies, biosensors, and thermoelectric and magnetic applications [31, 32, 33, 34, 35, 36, 37, 38, 39, 40]. Our group has demonstrated that key parameters—porosity, the number of transverse channels, and their spacing—can be accurately controlled [31, 41]. In addition, 3D‐AAO itself has been investigated as a radiative cooling material; however, to date, no polymer has been infiltrated into these templates to evaluate the cooling performance of the resulting composite material [42, 43].

Therefore, this study demonstrates, for the first time, the nanostructuring of PVDF via infiltration into 3D‐AAO nanoporous templates to produce a radiative cooling metamaterial. 3D‐AAO templates with a thickness of 40 μm, a transverse channel spacing of 200 nm, and an area of approximately 6 cm^2^ were fabricated for this purpose. After PVDF infiltration, three cooling methods were carried out to investigate the influence of the resulting polymer's phase proportion on its optical properties. In addition, two configurations were examined for each cooling method: 3D‐PVDF embedded in AAO and free‐standing 3D‐PVDF obtained after the chemical etching of the template to investigate how the optical response of the materials changes due to the different refractive indices of the filler (alumina or air).

Subsequently, a UV treatment was applied to whiten the 3D‐PVDF nanostructures via photo‐oxidation and further enhance its solar reflectance. Finally, the radiative cooling potential of the metamaterial was demonstrated experimentally through outdoor measurements conducted in Madrid between July and September 2025 under direct sunlight. It is important to note that this process is cost‐effective and scalable for industrial applications. This study presents a novel method for fabricating nanostructured PVDF within 3D‐AAO templates and demonstrates an efficient radiative cooler with potential for enhancing the energy efficiency of future cooling systems.

## 2 Results and Discussion

### 2.1 Morphological Characterization (SEM)

Cross‐sectional SEM images of the 3D‐AAO templates and 3D‐PVDF nanostructures are shown in Figure 1. The 3D‐AAO architecture (Figure 1a,c) consists of longitudinal nanochannels with a pore diameter of 46 ± 4 nm, interconnected by transverse channels with an average spacing of 202 ± 7 nm. The corresponding statistical distributions are presented in Figure 1b,d based on the analysis of three to five different regions per nanostructure to ensure structural uniformity. In Figure 1e, PVDF is observed to be fully filling the channels and overflowing from the AAO template in a nanostructured form. Finally, Figure 1f displays the 3D‐PVDF nanostructure after the removal of the AAO. No discernible morphological variations were observed among the different cooling methods, indicating that the cooling process does not compromise the structural integrity. These results confirm the successful fabrication of the 3D‐AAO templates, as well as the proper infiltration and nanostructuring of PVDF within the porous alumina template.

**FIGURE 1:**
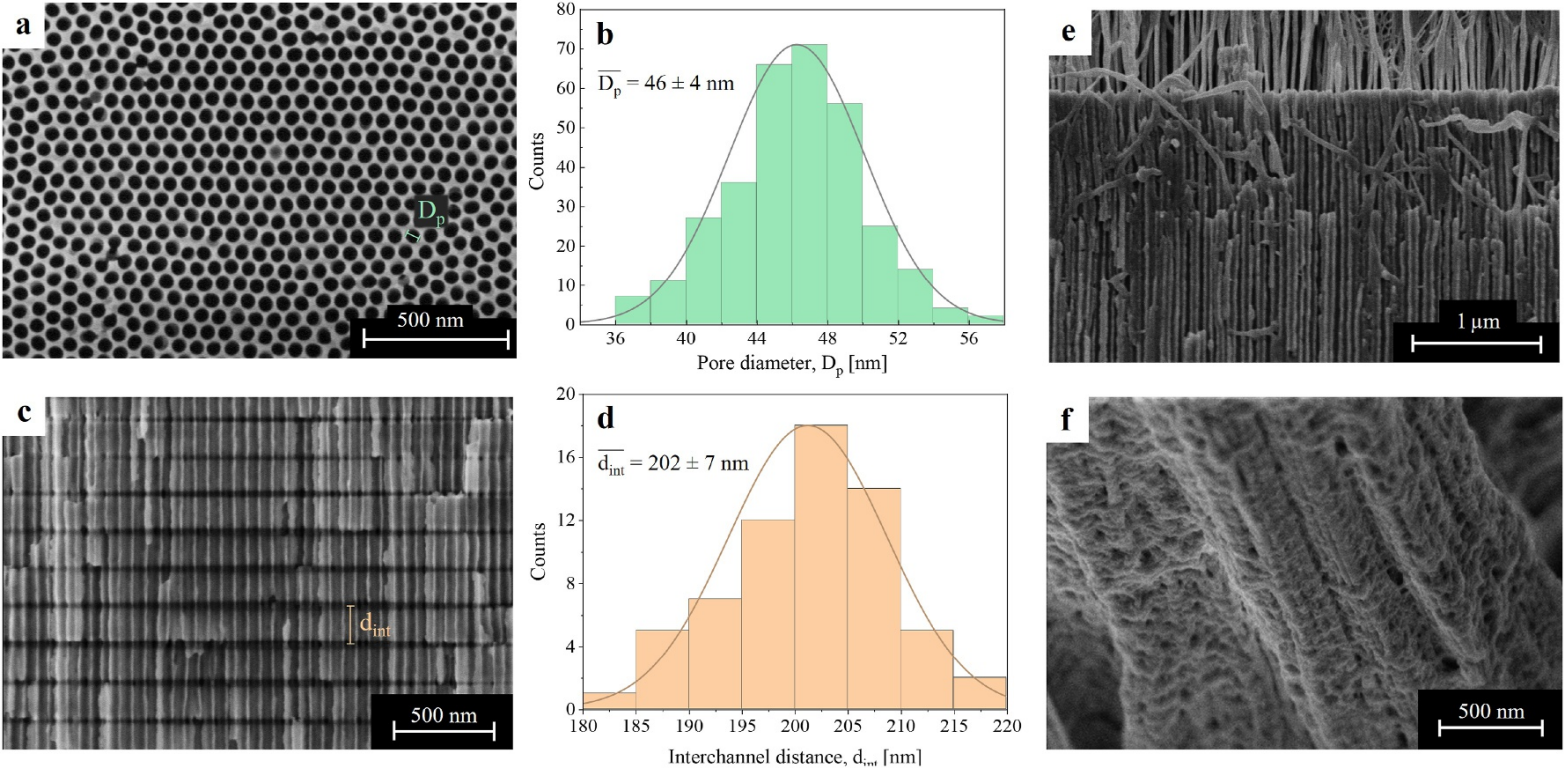
(a) Top‐view SEM image of the 3D‐AAO template, (b) pore diameter distribution, (c) cross‐sectional SEM image of 3D‐AAO, (d) interchannel spacing distribution, (e) cross‐sectional SEM image of 3D‐PVDF inside the AAO template, and (f) freestanding 3D‐PVDF nanostructure without AAO.

### 2.2 Chemical Characterization (ATR via FT‐IR)

ATR measurements were employed to characterize the chemical composition before and after UV treatment. Figure 2 presents the transmittance spectra of the initial PVDF film and the 3D‐PVDF with and without AAO, both before and after a 3‐day outdoor UV exposure. For the polymer film, no appreciable changes in chemical composition are observed following UV irradiation, suggesting that PVDF remains chemically stable under these conditions.

**FIGURE 2:**
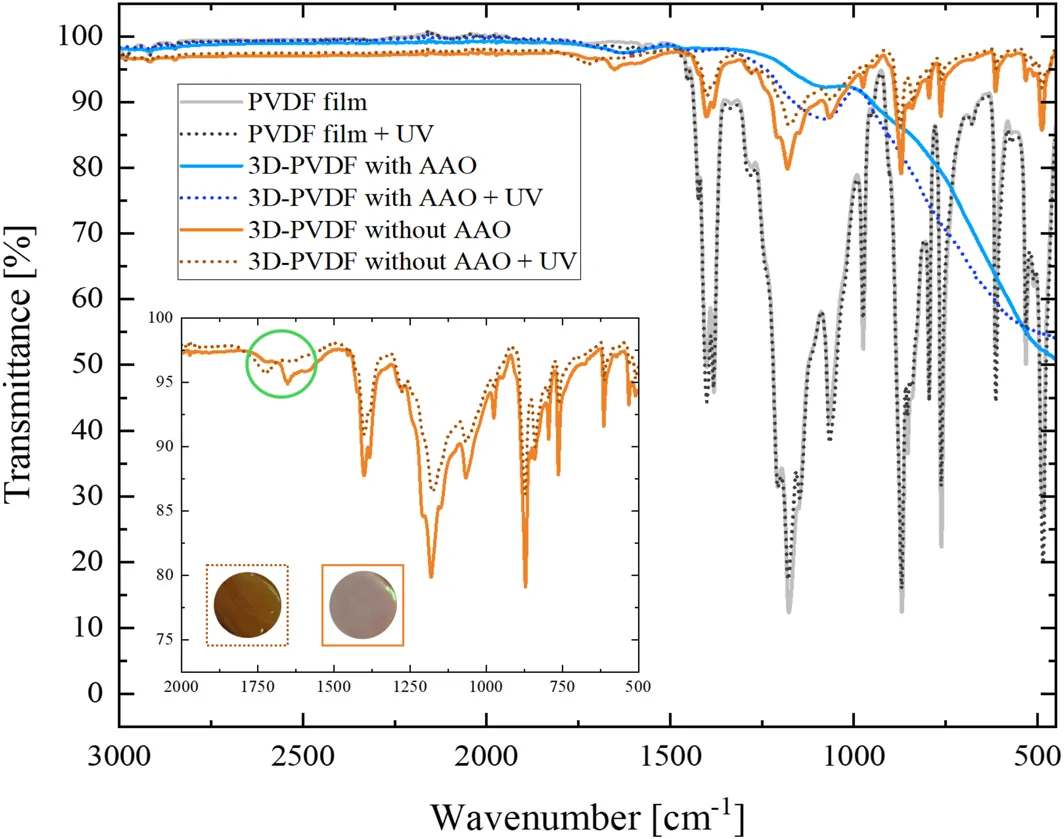
Transmittance spectra (baseline‐corrected) for a PVDF film (gray) and 3D‐PVDF without AAO (orange), before (dotted line) and after (solid line) the UV treatment.

In contrast, during infiltration into the AAO templates, changes in the polymer structure are observed, accompanied by the development of a brown coloration. The infiltration temperature (170°C) is slightly above the melting point of PVDF, where increased chain mobility allows the polymer to flow into the nanochannels. Under these conditions, the presence of structural defects promotes dehydrofluorination processes, leading to the formation of C=C bonds [44]. These double bonds can act as chromophores, which could explain the brown coloration of the nanostructured material, being consistent with the absorption band observed at approximately 1650 cm^−1^ for the 3D‐PVDF without AAO case (inset of Figure 2, circled in green). For the 3D‐PVDF with AAO configuration, the alumina matrix largely masks the polymer vibrational features due to the presence of *Reststrahlen bands*, resulting in the flattened spectra (blue curves) observed in Figure 2 [45].

Under UV exposure, the nanomaterial becomes progressively whiter and more translucent. The absorption spectra of both nanostructured PVDF before and after the UV treatment are almost identical, suggesting that no significant degradation occurs. The absorption spectra of the nanostructured PVDF before and after UV treatment remains largely similar, suggesting that no significant degradation of the polymer backbone occurs. However, the absorption band associated with C=C bonds is no longer observed after UV treatment, whereas a new band attributed to carbonyl (C=O) groups appears at approximately 1725 cm^−1^, highlighted in green in Figure 2 [46]. These observations suggest that UV irradiation promotes the photo‐oxidation of the defects formed during nanostructuring, thereby reducing the concentration of the chromophores responsible for the brown coloration [45].

Figure 3 shows the presence of different crystalline phases in the nanostructured PVDF, evidenced by their characteristic infrared absorption bands [47]. Only the AAO‐free PVDF cases are exhibited, since the presence of the AAO masks the underlying peaks of the polymer. The bands at 614 cm^−1^ and 713 cm^−1^ are associated with the *α*‐phase, whereas 1275 cm^−1^ corresponds to the *β*‐phase [48]. The absence of a discernible band at 1234 cm^−1^ suggests that the *γ*‐phase is barely present [49]. This observation is consistent with previous reports, which indicate that the *γ*‐phase tends to form at higher processing temperatures and for larger pore sizes when PVDF is confined within AAO templates [50].

**FIGURE 3:**
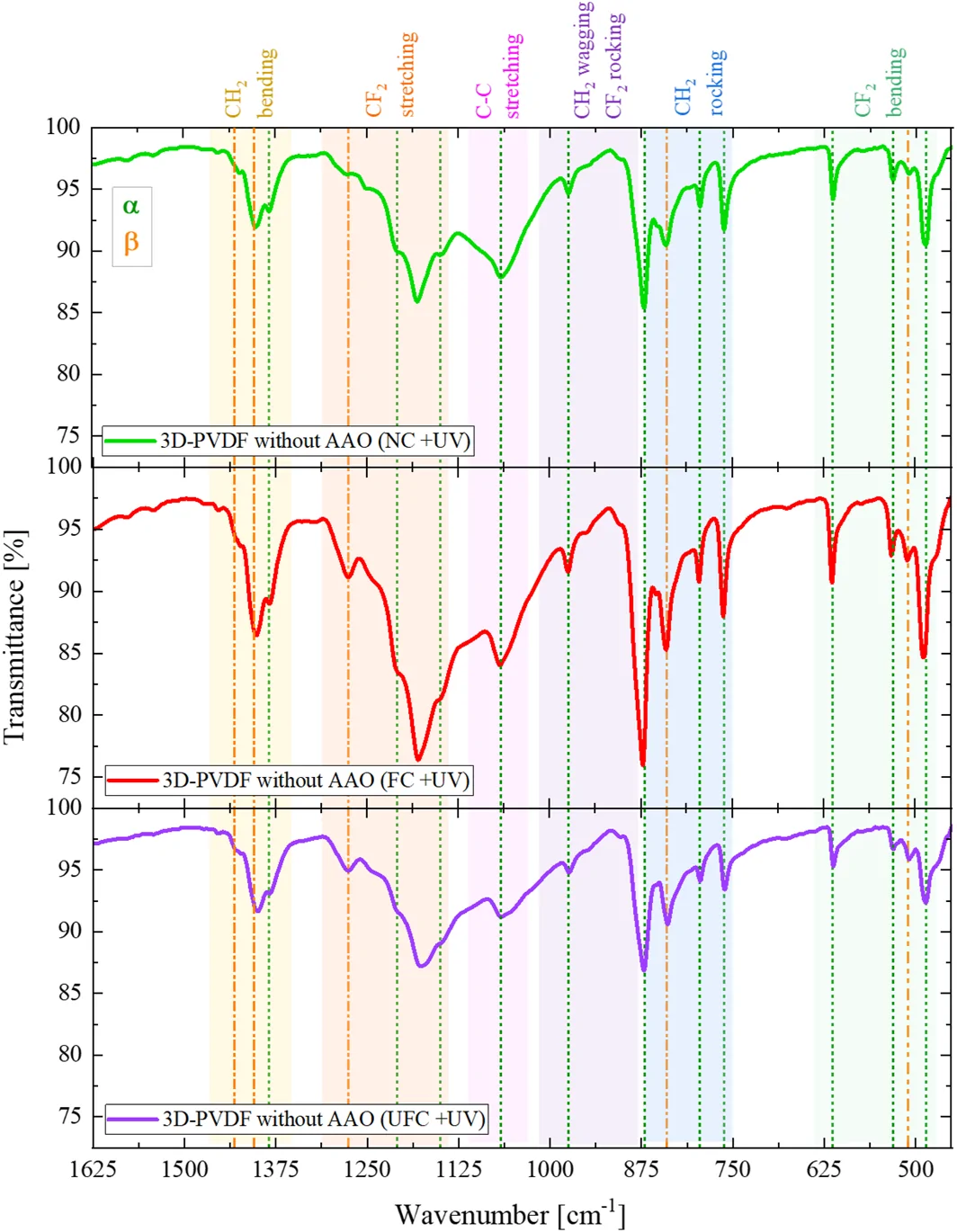
Transmittance spectra in the 450–1625 cm^−1^ range for the three cooling conditions of 3D‐PVDF without AAO. The characteristic absorption bands of PVDF's phases are highlighted in green and orange, whereas the vibrational mode ranges associated with the different chemical bonds are indicated by colored regions.

The relative fraction of the *β*‐phase, *F*(*β*), was estimated using the following expression:

$$F\left(\beta \right)=\frac{A\left(\beta \right)}{1.262\cdot A\left(\alpha \right)+A\left(\beta \right)\;},$$

where *A*(*α*) and *A*(*β*) are the absorbance values (*A =* 1−*T*) measured at 766 cm^−1^ and 840 cm^−1^, respectively [51]. Using this method, the *β*‐phase fractions, expressed as percentages, of the nanostructured PVDF subjected to natural, fast, and ultra‐fast cooling were 46.7% ± 0.6%, 49.8% ± 1.0%, and 54.4% ± 0.7%, respectively, with the associated errors calculated by propagation. The observed increase in the *β*‐phase content with increasing cooling rate is in good agreement with trends previously reported in the literature [52]. A higher content of the *β*‐phase has been associated with an increase in the polymer's refractive index, thereby enhancing the optical contrast between the nanostructure and the surrounding air, resulting in greater scattering of incident light according to Rayleigh theory [53]. Therefore, ultra‐fast cooling is the most suitable method for the PVDF‐based cooler fabrication.

### 2.3 Crystalline Structure (XRD—Phases)

Once the different crystalline phases present have been identified, the crystallization behavior of PVDF after nanostructuration was further investigated. Diffractograms of the nanostructured 3D‐PVDF, both with and without AAO, were collected for the three cooling rates and are presented in Figure 4, with the corresponding characteristic diffraction planes indicated [48].

**FIGURE 4:**
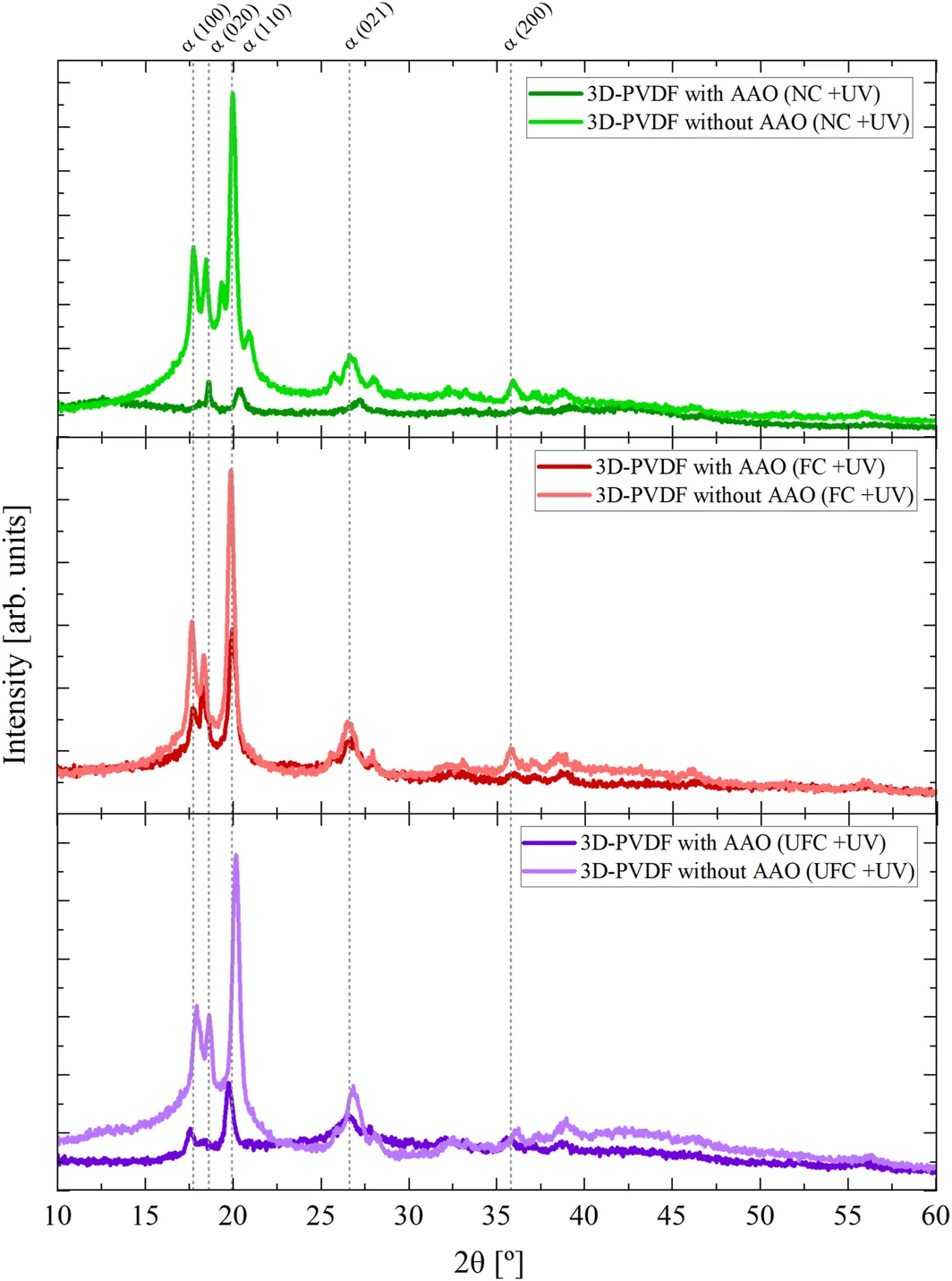
X‐Ray diffractograms of nanostructured 3D‐PVDF with and without AAO for the different cooling rates, with the characteristic peaks of the *α*‐phase marked.

The structural characterization of nanostructured PVDF is particularly challenging due to the overlap of diffraction peaks associated with coexisting crystalline phases at similar diffraction angles (*α* ∼ 19.9° and *β* ∼ 20.3°). This complexity is further compounded by small angular shifts, which may result from lattice distortions caused by nanostructuration, confinement within the AAO template, or the curvature of the material.

A notable trend observed in the diffractograms is a systematic shift of the diffraction peaks toward lower 2*θ* angles with increasing cooling rate. According to Bragg's law, this is indicative of an increase in interplanar spacing, suggesting an expansion of the crystalline lattice. This behavior is commonly associated with internal strain or lattice deformation [54]. During natural cooling, PVDF chains have more time to reorganize and adopt a more ordered packing, whereas faster cooling rates are likely to restrict chain mobility, thereby limiting efficient rearrangement and the formation of well‐defined crystalline domains.

Most of the observed peaks correspond to different planes of the *α*‐phase. In particular, the diffraction peaks observed at *2θ* ≈ 17.7°, 18.4°, 19.9°, 26.5°, 35.8°, and 38.6° correspond to the (100), (020), (110), (021), (200), and (131) planes of the *α*‐phase, respectively [52, 55]. The absence of the well‐defined characteristic planes of the *β*‐phase (110, 200) at 20.3° [48] suggests that this phase is not dominant. Nevertheless, minor contributions from the *β*‐phase cannot be excluded and may be associated with the weak diffraction peak observed at around 28.0°. In addition, diffractograms collected before and after the UV treatment for each cooling method exhibit only minor differences, as shown in Supporting Information S1: Figure S1–3, indicating that the UV exposure does not significantly alter the crystalline structure of the nanostructured PVDF.

Overall, the 3D‐nanostructured PVDF can be described as a semicrystalline material with polycrystalline ordered regions, predominantly composed of *α*‐phase crystallites with multiple crystallographic orientations, along with a smaller contribution from the *β*‐phase and an amorphous fraction.

### 2.4 Contact Angle—Wettability

Before analyzing the optical properties in detail, the surface wettability was assessed, as a previous study from our group showed that hydrophobic materials (e.g., cellulose networks) exhibit more stable cooling performance under varying humidity conditions than hydrophilic ones [10]. The wettability was evaluated by measuring the static water contact angle at three different locations on each PVDF nanostructure, both with and without the AAO template before and after the outdoor measurements (UV treatment). Values ranging from 90° to 100° with an experimental uncertainty of ± 5° were obtained in all cases, indicating hydrophobic surface behavior and self‐cleaning behavior are preserved (Supporting Information S1: Figure S4).

### 2.5 Optical Properties

To operate 24 h a day, a radiative cooler must present high reflectance across the visible solar spectrum and high emissivity in the infrared within the 8–13 μm atmospheric window. Since theoretical calculations of the cooling power density require knowledge of the material's spectral emissive behavior, the solar and infrared emissivities were determined from the measured reflectance data, assuming zero transmittance due to the optical opacity of the electropolished aluminum substrate incorporated underneath nanostructured 3D‐PVDF.

Figure 5 shows the overall emissivities of the different 3D‐PVDF nanostructures over the 0.35–17 μm wavelength range, together with the solar spectrum and atmospheric transmission spectrum [56, 57]. Regarding the solar emissivity (the emissivity in the solar range, 320–1200 nm), which should be as low as possible, the 3D‐PVDF nanostructures without AAO exhibit superior performance compared to those with AAO.

**FIGURE 5:**
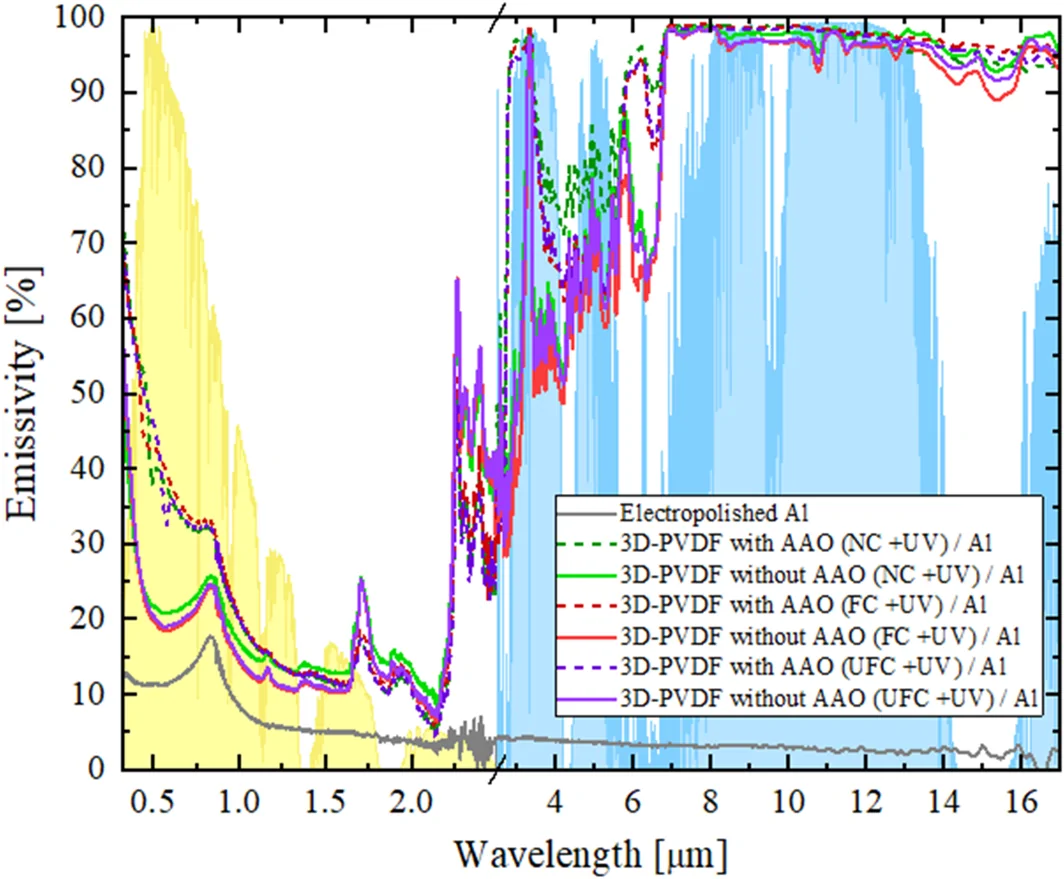
Emissivity spectra from the UV–Vis–NIR to the mid‐IR range of 3D‐PVDF nanostructures on an Al substrate, with and without AAO, prepared using different cooling methods. The solar spectrum is shown in yellow, and the atmospheric transmittance is shown in blue.

Specifically, the average solar emissivity ($\bar{\varepsilon_{\text{solar}}}$) ranges from 32.6% to 33.1% for 3D‐PVDF with AAO and from 17.6% to 21.1% for 3D‐PVDF without AAO. These values correspond to an average solar reflectance ($\bar{R_{\text{solar}}}$) of 66.9%–67.4% and 78.9%–82.4%, respectively, with the associated uncertainty of ± 0.5% estimated by error propagation from the spectral reflectance measurements. In both cases, ultra‐fast cooling yields the highest *R*_solar_ values, followed by fast and natural cooling, consistent with the phase composition observed for each method. The found value is lower than the existing solar reflectance values in the literature (see Supporting Information S1: Table S1) due to our 3D‐PVDF nanostructures being much thinner (40 μm) than hierarchically porous PVDF‐HFP [8, 24, 58], as well as composite systems incorporating inorganic fillers [59], which have thicknesses between 300 and 600 μm.

With respect to the infrared emissivity, all nanostructured 3D‐PVDF types exhibit very high values, with an average emissivity in the 8–13 μm range ($\bar{\varepsilon_{\text{IR}}}$) of 97.7%–98.4% for the nanostructures with AAO and 96.4%–97.7% for the nanostructures without AAO, with errors calculated from the experimental standard deviation (see Table 1). These values are similar to the values obtained for hierarchically porous PVDF‐HFP [8, 24, 58], as well as composite systems incorporating inorganic fillers [59] (see Supporting Information S1: Table S1). Moreover, the 3D‐AAO templating approach enables precise and reproducible control over the hierarchical polymer architecture, providing enhanced structural stability, tunable anisotropic properties, and a more reproducible methodology than other fabrication processes.

**TABLE 1:** Average solar reflectance ($\bar{R_{\text{solar}}})$ and infrared emissivity ($\bar{\varepsilon_{\text{IR}}})$ of the different 3D‐PVDF nanostructures, along with the theoretical cooling power density (*P*_cool_) and cooling temperature (Δ*T*) obtained during daytime and night‐time conditions.

			Night‐time (*T*_amb_ = 25°C)		Daytime (*T*_amb_ = 35°C)	
	$\bar{R_{\text{solar}}}$ (%) (0.32–1.2 μm)	$\bar{\varepsilon_{\text{IR}}}$ (%) (8–13 μm)	*P*_cool_ [W m^−2^]	Δ*T* [°C]	*P*_cool_ [W m^−2^]	Δ*T* [°C]
3D‐PVDF with AAO (NC + UV)/Al	67.4 ± 0.5	97.9 ± 0.9	87.1	5.6	84.0	4.7
3D‐PVDF without AAO (NC + UV)/Al	78.9 ± 0.5	97.7 ± 0.5	85.3	5.3	174.4	10.0
3D‐PVDF with AAO (FC + UV)/Al	66.9 ± 0.5	98.4 ± 0.5	87.2	5.6	79.7	4.5
3D‐PVDF without AAO (FC + UV)/Al	81.1 ± 0.5	96.4 ± 1.1	85.0	5.2	184.8	10.5
3D‐PVDF with AAO (UFC + UV)/Al	67.2 ± 0.5	98.1 ± 0.6	87.5	5.6	81.2	4.5
3D‐PVDF without AAO (UFC + UV)/Al	82.4 ± 0.5	96.7 ± 0.8	85.6	5.3	182.3	10.7

The present study focuses on template‐nanostructured PVDF architectures; planar PVDF films on metallic substrates and other polymer systems will be addressed in future studies. In conclusion, based on these emissivity values, a higher daytime cooling performance can be expected for the 3D‐PVDF without AAO nanostructures, particularly for the ultra‐fast cooling case, which exhibits a $\bar{R_{\text{solar}}}$ of 82.4% ± 0.5% and an $\bar{\varepsilon_{IR}}$ de 96.7% ± 0.8%. Additionally, the studies of solar reflectance enhancement with the UV treatment are shown in Supporting Information S1: Figures S5 and S6.

### 2.6 Cooling Power Calculations

To quantify the influence of the cooling rate on the radiative cooling performance, the cooling power density (*P*_cool_) was evaluated for both night‐time and daytime scenarios under representative atmospheric conditions. During night‐time, all nanostructures exhibit similar cooling behavior due to their nearly identical infrared emissivity, $\varepsilon_{\text{IR}}$. Considering an ambient temperature of 298 K (25°C), a *P*_cool_ of approximately 87 W m^−2^ is obtained, corresponding to a maximum achievable temperature reduction, Δ*T*, of ∼ 5.5°C (Figure 6a). Under these conditions, as expected, variations in cooling rate do not significantly affect the cooling performance. To avoid optimistic assumptions, a sensitivity analysis was performed by varying the convection coefficient, *h*_cc_ (0–12 W m^−2^ K^−1^), with the results presented in Supporting Information S1: Figure S7.

**FIGURE 6:**
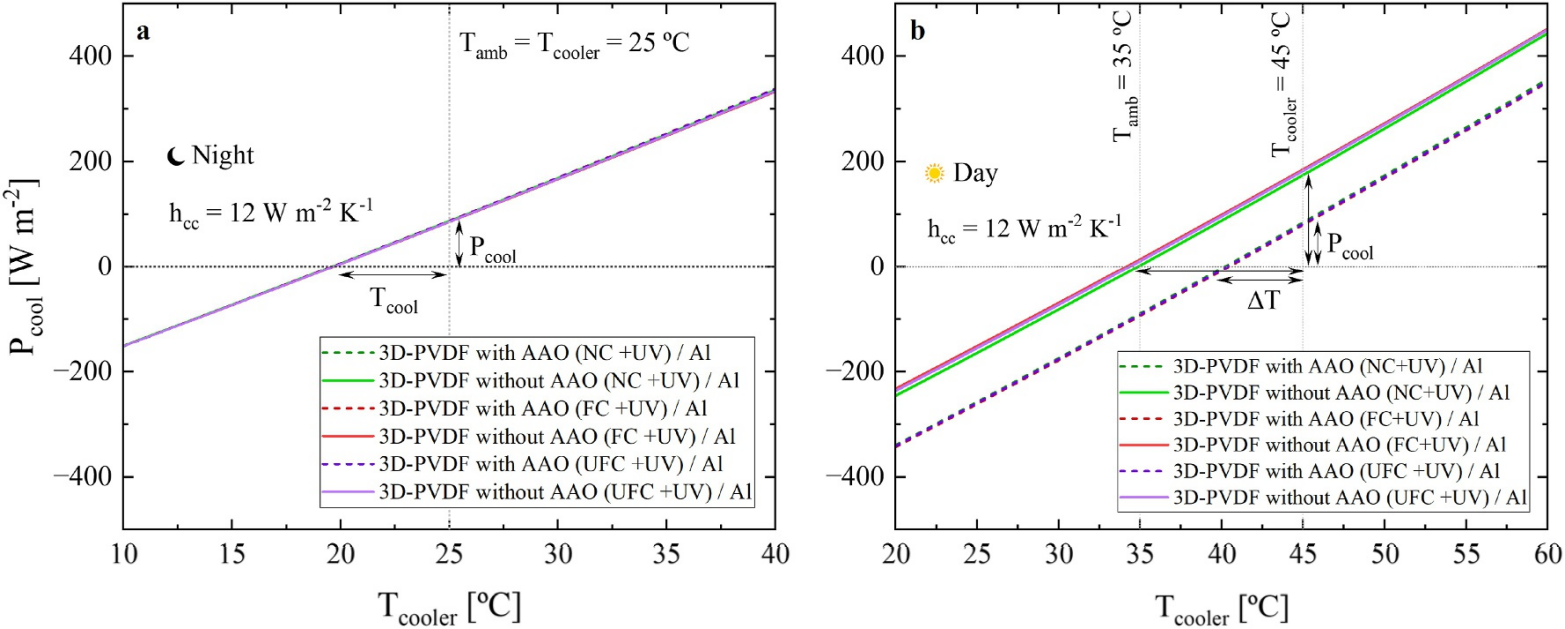
Cooling power density as a function of the cooler's temperature under (a) night‐time and (b) daytime conditions: 298 and 313 K, respectively, considering a *h*_cc_ of 12 W m^−2^ K^−1^.

During daytime, however, the calculations were performed assuming an ambient temperature of 35°C, representative of hot summer day conditions in Madrid. In addition, an initial cooler temperature of 45°C was selected to consider the solar heating prior to reaching thermal equilibrium and to reflect more realistic working conditions. Under these assumptions, differences in cooling performance become evident depending on whether the PVDF remains embedded within the AAO template or not (Figure 6b).

Nanostructured 3D‐PVDF with AAO, at different cooling rates, can achieve a *P*_cool_ of ∼ 80 W m^−2^, with a temperature reduction of approximately 4.5°C. In contrast, nanostructured 3D‐PVDF without AAO exhibits superior cooling performance, reaching values of approximately 180 W m^−2^ and Δ*T* up to 10.8°C. This enhanced performance can be primarily attributed to the lower effective solar emissivity (i.e., higher solar reflectance) of the AAO‐free nanostructures, which reduces solar heat absorption during daytime. It should be noted that these values represent theoretical limits under certain conditions. In practical outdoor environments, factors such as temperature fluctuations, variations in solar irradiance, and relative humidity, among others, are expected to influence the actual cooling performance. Moreover, these calculations only consider radiative transfer, together with a moderate convective contribution. However, under real conditions, additional conductive heat exchange with the surroundings may happen, affecting the resulting Δ*T* and *P*_cool_.

Table 1 summarizes the principal magnitudes used to describe and compare the different 3D‐PVDF coolers, predicting a better performance for the free‐standing 3D‐PVDF, which is consistent with the analysis presented in the previous section.

Comparing the calculated cooling power density with values reported in other studies results challenging, as this parameter depends on several variables, including the selected atmospheric emissivity, the emissivity range considered, and the assumed ambient and cooler temperatures. Moreover, most previous PVDF‐based radiative coolers are combined with other materials and employ different geometrical configurations, which further complicates direct comparisons. Nevertheless, the order of magnitude of the calculated *P*_cool_ values is within the same range to those reported in the literature, such as 148.3 W m^−2^ for PVDF composite with Al_2_O_3_ fillers and 137 W m^−2^ for nanoporous PVDF coating film made with a ZIF8 MOF sacrificial template [18, 60].

### 2.7 Outdoor Measurements

Outdoor characterization was carried out over several summer days under varying atmospheric conditions to evaluate the real cooling performance of 3D‐PVDF nanostructures on top of an aluminum bulk. Figure 7 illustrates the outdoor temperature profiles of the 3D‐PVDF nanostructures on the aluminum substrate measured over three different days between July and September 2025, with the empty box used as a reference (its optical properties are presented in Supporting Information S1: Figure S8).

**FIGURE 7:**
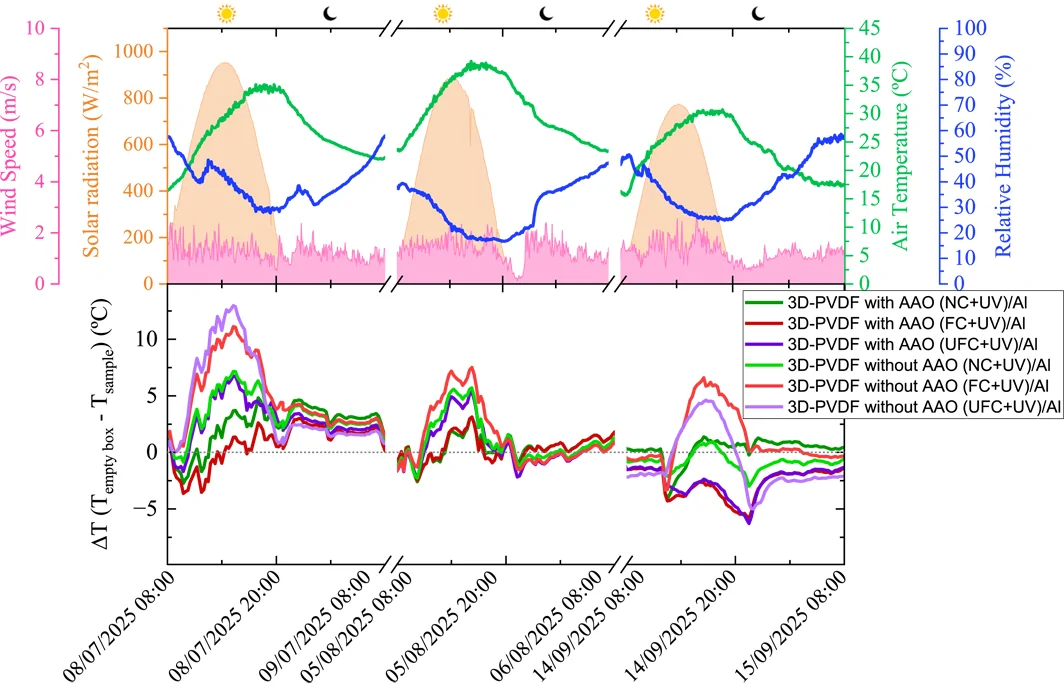
Outdoor passive radiative cooling characterization, including the weather conditions during the measurements and the resulting temperature reduction calculated with respect to both references, for 8–9 July, 5–6 August, and 14–15 September 2025.

In addition, Table 2 summarizes the daytime and night‐time temperature reductions for different days (8–9 July, 5–6 August, and 14–15 September) of different measurements periods, relative to the empty box. The associated uncertainty was estimated to be ± 0.7°C by error propagation.

**TABLE 2:** Temperature difference, Δ*T*, relative to the empty box for different summer days in 2025 under different atmospheric conditions (uncertainty ± 0.7°C).

	8–9 July, 2025		5–6 August, 2025		14–15 September, 2025	
	*T* = 27°C, *H* = 31%	*T* = 35°C, *H* = 39%, RS = 930 W m^−2^	*T* = 25.4°C, *H* = 36.5%	*T* = 38.4°C, *H* = 18%, RS = 802 W m^−2^	*T* = 20°C, *H* = 42.6%	*T* = 30°C, *H* = 26%, RS = 614 W m^−2^
	Δ*T*_night_ [°C]	Δ*T*_day_ [°C]	Δ*T*_night_ [°C]	Δ*T*_day_ [°C]	Δ*T*_night_ [°C]	Δ*T*_day_ [°C]
3D‐PVDF with AAO (NC + UV)/Al	4.0	3.7	0.9	3.1	0.9	1.4
3D‐PVDF with AAO (FC + UV)/Al	2.5	1.4	0.9	3.1	—	—
3D‐PVDF with AAO (UFC + UV)/Al	2.8	6.8	—	5.3	—	—
3D‐PVDF without AAO (NC + UV)/Al	3.4	7.2	0.2	5.7	0.3	0.9
3D‐PVDF without AAO (FC + UV)/Al	3.5	11.1	—	7.5	0.3	6.6
3D‐PVDF without AAO (UFC + UV)/Al	2.2	12.9	*	*	—	4.6

*Note:* * = data unavailable due to thermocouple failure, Dash (–) = no cooling observed.

Focusing first on the measurements conducted on 8–9 July 2025, the sky remained clear, which can be appreciated by the smooth solar radiation curve. The highest temperature, 36.3°C, and lowest humidity, 27%, happened at around 17:30, whereas the maximum solar radiation, 962 W m^−2^, was reached around 14:30. During night‐time, the temperature gradually decreased until it reached its minimum value at 6:30, whereas the relative humidity increased until values around 64%, at the same hour. This behavior is consistent with typical summer conditions in Madrid. Under these environmental conditions, the temperature inside the empty reference box varied from approximately 21°C at night to 55°C under maximum solar radiation, whereas the aluminum covered box exhibited lower temperatures, ranging from about 20°C at night to 50°C during the daytime under the same conditions. Clear differences in cooling performance are observed between daytime and night‐time operation. During daytime, the 3D‐PVDF nanostructures without AAO exhibit greater cooling compared to those with AAO, achieving temperature reductions of approximately 7°C–13°C, whereas the PVDF nanostructures with AAO showed reductions of 1.5°C–6.8°C. This daytime cooling response is particularly relevant for real‐world applications, as it coincides with peak solar irradiance, when cooling demand is highest.

Among the AAO‐free PVDF nanostructures, the ultra‐fast cooled 3D‐PVDF exhibits the best performance, with a temperature reduction of 12.9°C, relative to the empty box, followed by the fast cooled (11.1°C), and the natural cooled (7.2°C) nanostructures. This trend is consistent with their optical properties shown in Figure 5. It is also worth noting that larger temperature reductions are observed when the PVDF nanostructures are referenced to the uncoated empty box than to the aluminum‐covered box, as the higher solar reflectance of the aluminum surface mitigates solar heating.

In contrast, during night‐time, the trend reverses, with the 3D‐PVDF nanostructures with AAO showing greater cooling (2.5°C–4.0°C) than those without AAO (2.2°C–3.5°C). Nevertheless, the night‐time cooling behavior is more uniform because in the absence of solar radiation, the heat transfer is governed by the mid‐infrared emissivity, which is similar for the different PVDF nanostructures.

On 8 July 2025, at the moment of maximum cooling, the highest solar irradiance among the three days shown in Figure 7 was recorded (930 W m^−2^), and the differences between the nanostructures were more pronounced, as daytime performance is strongly influenced by solar reflectance. This day also exhibited the largest Δ*T* for all PVDF nanostructures since the polystyrene reference absorbed more radiation and therefore reached higher temperatures. In contrast, 5 August 2025 was hotter (38.4°C) and dry (18%) but presented lower maximum solar irradiance (802 W m^−2^). As a result, the reference absorbed less solar energy compared to 8 July, and its temperature remained closer to that of the nanostructures, leading to smaller relative temperature differences. Lastly, 14 September 2025 presented the lowest irradiance (614 W m^−2^), and the corresponding temperature reductions were very minimal or almost negligible.

During night‐time, cooling is mainly governed by infrared emission. Increased humidity reduces the atmospheric transparency window in the 8–13 μm range, limiting the amount of heat that can be radiated to space [61]. For this reason, the clear and dry night of 8 July resulted in noticeable cooling for all nanostructures. In contrast, the most humid and cooler nights in August and September limited the cooling effect, with no observable cooling as the nanostructures and reference reached similar temperatures.

In summary, nanostructured PVDF has demonstrated a robust and reliable capacity to provide cooling under a wide range of atmospheric conditions. Its performance is enhanced during daytime under higher solar irradiance and during night‐time when ambient temperatures are elevated. This adaptive behavior underscores its performance under peak cooling demand, highlighting its potential as a scalable and energy‐efficient passive cooling strategy.

To assess long‐term stability, transmittance spectra were performed before and after UV treatment of 3D‐PVDF nanostructures (see Figure 2). This confirmed that there were no further chemical changes to the polymer backbone. The water contact angle remained in the 90°–100° range before and after outdoor measurements (UV treatment). There was no evidence of embrittlement or cracking after outdoor measurements (UV treatment) (see Video S1 of this process in the Supporting Information).

The manufacturing costs of these 3D‐PVDF nanostructures were estimated based on the production price reported in the reference [62] for AAO nanostructures, with the actual metal (aluminum) and polymer (polyvinylidene fluoride) prices taken into account. The relative cost of fabricating 3D‐PVDF nanostructures is 8.8·10^−5^ €/cm^2^ for low‐purity aluminum and 5.8·10^−3^ €/cm^2^ for high‐purity aluminum. These results indicate that 3D‐PVDF nanostructures are susceptible to mass production using inexpensive raw materials commonly used in industry, since conventional alumina templates are produced industrially in m^2^ and our process can easily be implemented in production lines.

## 3 Conclusions

In summary, nanostructured 3D‐PVDF‐based radiative coolers were successfully fabricated using 3D‐AAO templates and engineered through controlled post‐infiltration cooling methods. The cooling rate modulated the *α*/*β* crystalline phase ratio of PVDF, which influenced the optical response of the different nanostructures with and without AAO. A UV post‐treatment of 96 h under direct sunlight was applied to enhance solar reflectance via a photo‐oxidation process that whitens the nanomaterial without detectable degradation. The optical properties of 3D‐PVDF without AAO were found to be more favorable for daytime cooling applications than those of 3D‐PVDF with AAO. Specifically, free‐standing 3D‐PVDF that underwent ultra‐fast exhibited the highest *β*‐phase fraction (54.4% ± 0.7%) for optimal scattering, achieving an average solar reflectance of 82.4% ± 0.5% and IR emissivity of 96.7% ± 0.8%. Theoretical cooling power calculations indicate that significant temperature reductions could be achieved under representative conditions given their optical behavior, with a maximum value of ∼ 182 W m^−2^ for 3D‐PVDF without AAO (UFC) under standard solar irradiance. Outdoor measurements confirmed these predictions, with the highest temperature reductions of 12.8°C observed for UFC cooled 3D‐PVDF without AAO on hot, dry days, relative to the empty box. Notably, both optical characterizations and outdoor measurements were performed with an aluminum substrate beneath the nanostructured material. Overall, the results demonstrate the versatility of AAO templates for nanostructuring other polymers. Key advantages include the ability to tune the optical properties by varying the template geometry, the combination of sufficient rigidity for infiltration with flexibility after polymer removal, the hydrophobicity for humidity stability and the scalability of the material for diverse applications such as building and electronics cooling, and personal thermal management, among others.

## 4 Methods

### 4.1 Fabrication of Nanostructured PVDF Inside 3D‐AAO Templates

The first stage of the radiative cooler fabrication involved the production of the 3D‐AAO, using a two‐step anodization process [31, 35, 41, 63, 64]. High‐purity aluminum foil (99.999%, Advent Research Materials) was first cleaned in an ultrasonic bath with solvents of varying polarity (acetone, distilled water, isopropanol, and ethanol). The substrate was then electropolished in an aqueous solution of perchloric acid (HClO_4_) and ethanol (1:3) at 0°C under 20 V for 4 min to reduce surface roughness.

A first anodization was carried out in a 0.3‐M sulfuric acid (H_2_SO_4_) aqueous solution at 0°C under a constant voltage of 25 V for 24 h, resulting in the formation of an alumina layer with disordered longitudinal channels. This layer was subsequently removed by chemical etching in aqueous solution containing chromic oxide (Cr_2_O_3_, 1.8 wt.%) and phosphoric acid (H_3_PO_4_, 7 wt.%) for 24 h at room temperature, leaving the aluminum substrate with a patterned surface. Next, a second pulsed anodization was performed under alternating mild (25 V) and hard (33 V) conditions for 8 h 20 min, resulting in the formation of a 40.0 ± 0.4 μm thick AAO layer. The pulse duration was set to 2 s at hard voltage (33 V) to initiate the transverse channels, whereas the resting time at mild voltage (25 V) was adjusted to 180 s to obtain an average interchannel distance ($\bar{d_{\mathrm{int}}}$) of ≈ 200 nm. The remaining aluminum foil was then removed with an aqueous solution containing copper (II) chloride (CuCl_2_, 11.2 g), hydrochloric acid (HCl, 33.45 mL), and distilled water (H_2_O, 500 mL). Finally, aqueous solutions of H_3_PO_4_ (10 wt.% and 5 wt.%) at 30°C were employed to remove the barrier layer and open the transverse channels, respectively. Following this procedure, a 3D‐AAO template with an average pore diameter ($\bar{D_{p}}$) of 46 ± 4 nm, an interpore distance ($\bar{D_{\mathrm{int}}}$) of 67 ± 4 nm, and an interchannel distance ($\bar{d_{\mathrm{int}}}$) of 202 ± 7 nm was obtained.

The second stage of the radiative cooler fabrication process (Figure 8a) involved the infiltration of a 100‐μm thick translucent PVDF film (Goodfellow) into the 3D‐AAO template using a vacuum oven under a pressure of 80 mbar. Prior to infiltration, thermal analyses of the polymer were conducted to determine the suitable working temperature range. Differential scanning calorimetry (DSC, TA Instruments DSC‐Q100) revealed a crystallization temperature of 140.8°C and melting temperatures of 145.4°C and 163.5°C for the *α*‐ and *β*‐phases of PVDF, respectively (Supporting Information S1: Figure S9) [50, 65]. Furthermore, thermogravimetric analysis (TGA, TA Instruments Thermogravimetric Analyzer Q500) showed that polymer decomposition begins at approximately 485°C (Supporting Information S1: Figure S10).

**FIGURE 8:**
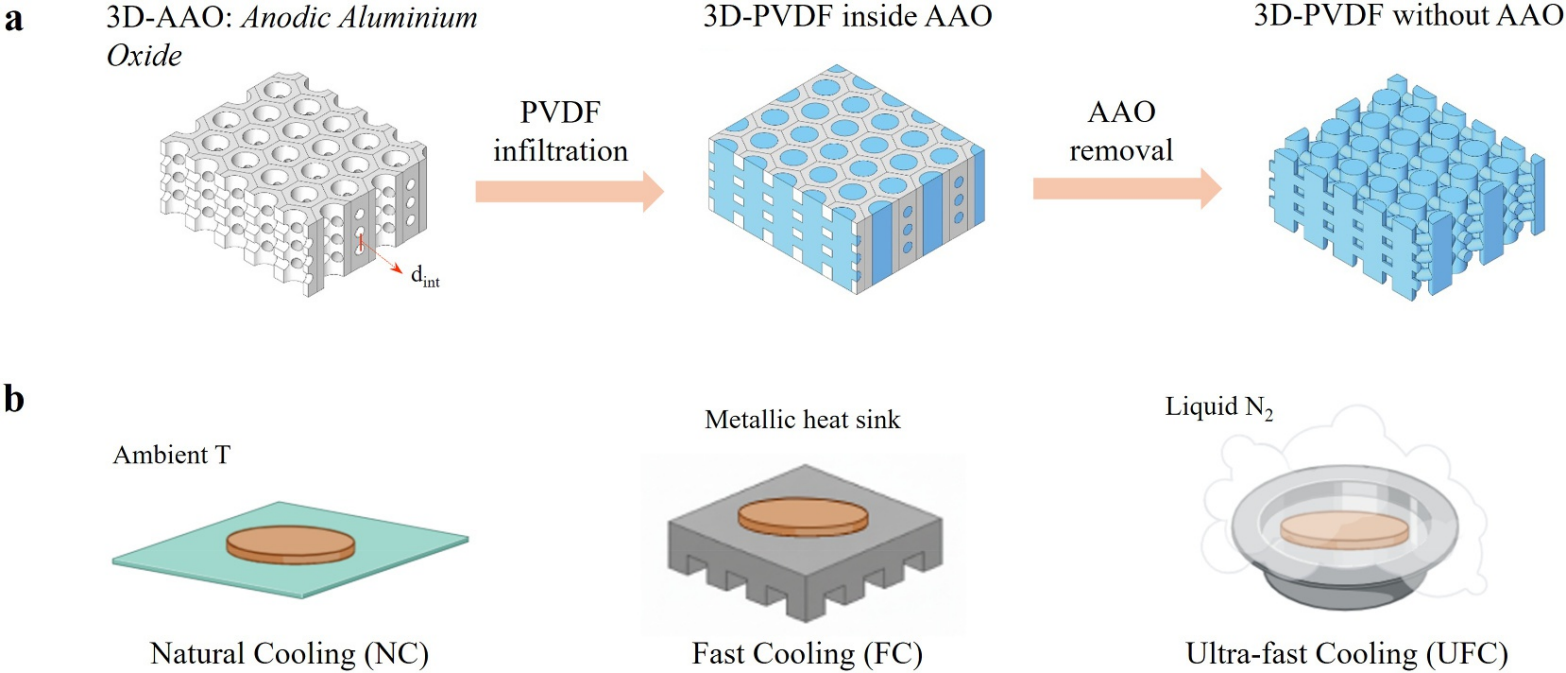
(a) Schematic illustration of the fabrication steps for nanostructured PVDF inside 3D‐AAO templates. (b) Schematic representation of the different cooling methods applied.

Based on these results, several infiltration temperatures (135°C, 145°C, 155°C, and 165°C) and durations (4, 8, 12, 16, and 24 h) were tested to determine the optimal infiltration conditions. The best results were obtained at 165°C and 24 h. Consequently, PVDF nanostructuring was performed by placing the polymer film onto the AAO in a preheated oven at 165°C under a vacuum of 80 mbar for 24 h. Additionally, a chemical etching step using an aqueous solution of H_3_PO_4_ (10 wt.%) was applied to remove the alumina, leaving only the nanostructured 3D‐PVDF. This approach enables the comparison of two configurations (Figure 8a) with distinct refractive index contrast between the components: polymer and alumina in the case of 3D‐PVDF with AAO and polymer and air in the free‐standing 3D‐PVDF without AAO.

Furthermore, to investigate the influence of PVDF crystalline phases on the optical and thermal response, three cooling procedures were applied at the end of the infiltration process: natural cooling (NC), fast cooling (FC), and ultra‐fast cooling (UFC) (Figure 8b). Briefly, in the NC method, the nanostructured 3D‐PVDF was allowed to cool gradually to room temperature inside the switched‐off oven. In the FC method, the sample was removed from the oven and immediately placed in contact with a metallic heat sink maintained at −15°C. Finally, in the UFC method, the 3D‐PVDF was immersed in liquid nitrogen for 10 s immediately following infiltration.

After cooling, the nanostructured 3D‐PVDF underwent a UV treatment to induce photo‐oxidation, whitening the material and enhanced its solar reflectance. Various exposure times (1, 2, 4, 8, 16, 24, 48, and 96 h) were tested using an Insolator IF‐48 (FREAK) with an irradiance of 10 mW cm^−2^. With increasing exposure, the reflectance gradually increased, accompanied by a progressive fading of the nanomaterial's color, resulting in a more translucent appearance. Finally, an exposure of 96 h under natural sunlight was selected to achieve the maximum whitening effect. Figure 9 shows the appearance of the 3D‐AAO template (Figure 9a), which acts as a Bragg reflector, and the 3D‐PVDF before (Figure 9b) and after the UV treatment (Figure 9c).

**FIGURE 9:**
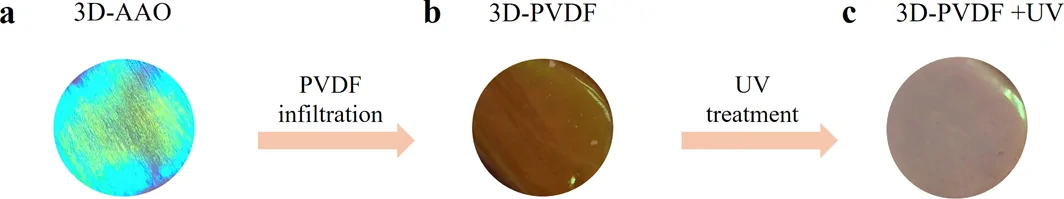
Images of (a) a 3D‐AAO template, (b) 3D‐PVDF with AAO before, and (c) after the solar UV treatment.

### 4.2 Morphological, Chemical, Structural, and Optical Characterization of PVDF Nanostructures Inside 3D‐AAO Templates

The morphological characterization of both the 3D‐AAO templates and 3D‐PVDF nanostructures was performed using high‐resolution field emission scanning electron microscopy (*FE*‐*SEM,* FEI VERIOS 460), operated at 2 kV and 13 pA. The presence and proportion of the existing phases were analyzed via attenuated total reflectance spectroscopy (ATR), using a Fourier transform infrared spectrometer (Perkin Elmer Frontier Spectrum 3) equipped with an ATR module, with an associated measurement uncertainty of approximately ± 2%. Additionally, the crystallization behavior was investigated using X‐ray diffraction (XRD, Bruker D8 Discover), with an associated *2θ* measurement uncertainty of ± 0.02°. The hydrophobicity of the nanostructures was studied through contact angle measurements performed with a Drop Shape Analyzer DSA25S (Krüss). The measurements were performed at room temperature, and the associated experimental uncertainty is estimated at ± 3°.

Regarding the optical properties, a UV–Vis–NIR PerkinElmer Lambda 950 double‐beam spectrophotometer equipped with a 150‐mm Spectralon‐coated integrating sphere was used to measure solar reflectance in the 0.3–2.5 μm range, with an associated measurement uncertainty of ± 0.5%. These values are presented in this work along with a solar spectrum obtained under AM 1.5 G conditions, measured by the National Renewable Energy Laboratory (NREL) [56]. Infrared reflectance (2.5–17 μm) was obtained using a gold integrating sphere module connected to the FT‐IR spectrometer (Perkin Elmer Frontier Spectrum 3), with an estimated measurement uncertainty of ± 1.5%. Both optical measurements were conducted with a 500‐μm thick aluminum foil beneath the 3D‐PVDF nanostructures, as this metal exhibits high reflectance across the UV–Vis and NIR–MIR spectral ranges [66]. The infrared emissivity ($\varepsilon_{\text{IR}})$ was obtained as 1−*R*, since the aluminum is optically opaque and transmission within this range is negligible. The emissivity values are presented alongside atmospheric transmission data obtained from the United Kingdom Infrared Telescope (UKIRT) website [57].

Unless otherwise stated, each key fabrication condition was repeated on at least three independent samples, and optical spectra were measured at different positions per sample to capture spatial variability.

### 4.3 Radiative Cooler Performance of PVDF Nanostructures Inside 3D‐AAO Templates

The radiative cooling power densities of the 3D‐PVDF nanostructures were calculated using the equations established for heat exchange between the cooler and its surroundings (see Supporting Information S1: Equations S1–S7) [3, 67]. For the night‐time scenario, an ambient temperature (*T*_amb_) of 298.15 K was assumed, whereas for the daytime case, a value of 313.15 K was considered, reflecting a representative peak summer temperature in Madrid [43]. In both scenarios, a convective heat transfer coefficient of 12 W m^−2^ K^−1^ was adopted to represent moderate natural convection [68]. The maximum temperature reduction (Δ*T*) was determined as the difference between the ambient temperature and the cooler's equilibrium temperature, *T*_cool_, corresponding to the steady‐state condition at which the net cooling power is zero. These values represent the theoretical limit of cooling that the material could achieve under the defined conditions. However, it is important to note that real environmental conditions present daily fluctuations in ambient temperature, solar radiation, relative humidity, and wind speed, among other factors, which can significantly influence the overall performance [69].

Lastly, outdoor cooling measurements of the 3D‐PVDF nanostructures were carried out on the rooftop of the IMN‐CSIC in Tres Cantos, Madrid (40.5920° N, 3.7080° W), using the experimental setup shown in Figure 10a, previously reported in ref. [67]. The nanostructures were placed in polystyrene boxes (Figure 10b) that acted as thermal insulators. These boxes were covered with aluminum foil to reflect incident sunlight and sealed with a low‐density polyethylene (LDPE) film to minimize nonradiative heat exchange with the surroundings. An aperture was made in each box to insert a thermocouple in direct contact with the material, with an associated measurement uncertainty of ± 0.5°C. The boxes were then mounted on a wooden platform inclined at 30° toward the south. An empty polystyrene box was used as the reference to enable meaningful comparisons with previous studies, as it is commonly adopted as a reference material in the literature [67, 70]. In addition, a weather station was installed adjacent to the experimental setup to monitor the atmospheric conditions during the measurements, including ambient temperature, solar irradiance, and relative humidity.

**FIGURE 10:**
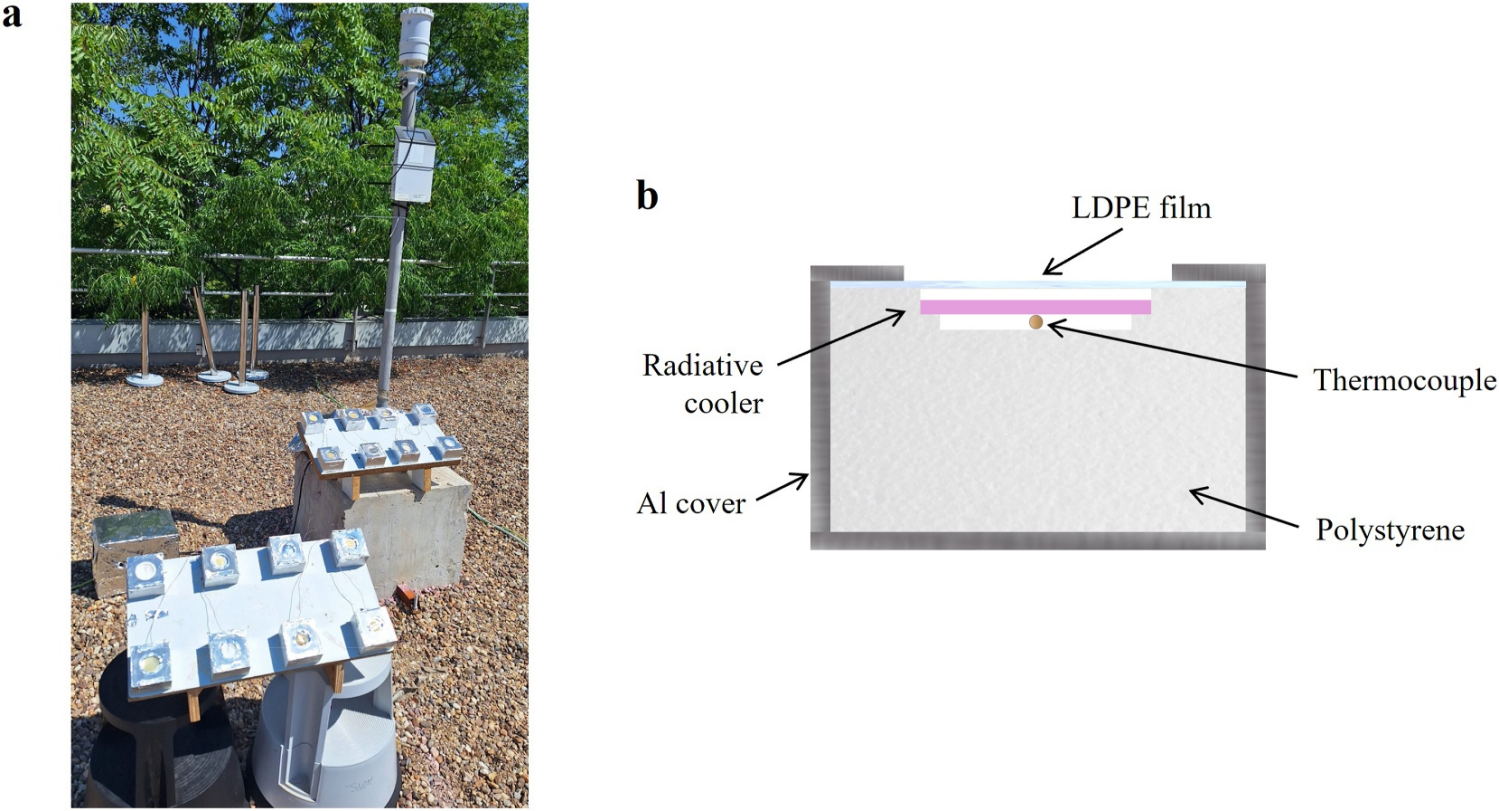
(a) Outdoor experimental set‐up on the rooftop in Tres Cantos, Madrid, Spain, with the weather station visible in the background. (b) Schematic representation of the empty polystyrene box.

## Author Contributions

**Amaia Iglesias‐Elcano:** conceptualization, investigation, writing – original draft, writing – review and editing, visualization, methodology, validation, formal analysis, data curation. **Luis Moreno‐Sanabria:** investigation, writing – original draft, writing – review and editing, visualization, validation, methodology, formal analysis, data curation. **Marisol Martín‐González:** methodology, validation, visualization, writing – review and editing, writing – original draft, formal analysis. **Cristina V. Manzano:** conceptualization, investigation, funding acquisition, writing – original draft, writing – review and editing, visualization, validation, methodology, project administration, formal analysis, data curation, supervision, resources.

## Conflicts of Interest

The authors declare no conflicts of interest.

## Supporting information


Supporting Information S1

**Video S1:** The free‐standing 3D‐PVDF nanostructures retained their mechanical flexibility after outdoor testing (UV treatment), with no evidence of embrittlement or cracking.

## Data Availability

The data supporting this article have been included as part of the article or Supporting Information S1.
