# Supplementary material for: Template‐Assisted 3D‐PVDF Nanonetworks for Passive Daytime Radiative Cooling
Source: Nanophotonics. 2026 Jun 29;15(13):e70183. doi: 10.1002/nap2.70183 (PMC13642497; doi:10.1002/nap2.70183)
Supplement: Supplementary file 1 [file NAP2-15-e70183-s002.docx]

**Supporting Information**

**Template-Assisted 3D-PVDF Nanonetworks**

**for Passive Daytime Radiative Cooling**

Amaia Iglesias-Elcano^*^, Luis Moreno-Sanabria^*^, Marisol Martín-González^*^, Cristina V. Manzano^*^

^*^Instituto de Micro y Nanotecnología, IMN-CNM, CSIC (CEI UAM+CSIC) Isaac Newton, 8, E-28760, Tres Cantos, Madrid, Spain

**Figure S1** XRD diffractograms of 3D-PVDF with and without AAO for natural cooling (NC), before and after the UV treatment (purple and green, respectively).

**Figure S2** XRD diffractograms of 3D-PVDF with and without AAO for fast cooling (FC), before and after the UV treatment (blue and yellow, respectively).

**Figure S3** XRD diffractograms of 3D-PVDF with and without AAO for ultra-fast cooling (UFC), before and after the UV treatment (red and orange, respectively).

**Figure S4** Optical images of the contact angles created in the interface between a water drop and the 3D-PVDF nanostructures before and after outdoor measurements (UV treatment), with an experimental uncertainty of ± 5 º.

**Table S1** Solar reflectance (*R_solar_*) and infrared emissivity (ε_IR_) of hierarchically porous PVDF-HFP as well as composite systems incorporating inorganic fillers.

|  | *R_solar_* (%)  (0.32 – 1.2 µm) | ε_IR_ (%)  (8 -13 µm) | Thickness | Reference |
| --- | --- | --- | --- | --- |
| Hierarchically porous P(VDF-HFP) | 96 ± 3 | 97 ± 2 | 300 µm | [1] |
| Hierarchically aligned porous PVDF | 97 | 96 | - | [2] |
| Hierarchically porous P(VDF-HFP) with Al_2_O_3_ microspheres | 98.26 | 97.56 | 559 µm | [3] |
| P(VDF-HFP) and Al_2_O_3_ | 97.5 | 94.8 | 570 µm | [4] |

**Figure S5** Reflectance evolution as a function of the UV lamp time exposure for the 3D-PVDF with AAO (NC) case, with an aluminium substrate underneath.

Figure S6 shows the reflectance of the nanostructured PVDF with and without the AAO template, before and after different UV exposure, under the different cooling conditions. As a reference, the solar spectrum over the same wavelength range is also included. Within the visible wavelength range (380–750 nm) a significant increase in reflectance is observed. In contrast, the emissivity in the infrared region remains unaffected by UV irradiation.

**Figure S6** 3D-PVDF solar reflectance before and after UV treatment for the three cooling cases, with the corresponding optical images of the samples on the right.

For nanostructured PVDF with AAO, the average solar reflectance initially ranges from 33.1 % to 37.4 %, increasing to 66.9 – 67.4 % after the treatment. In contrast, nanostructured PVDF without AAO exhibits higher reflectance values of approximately 78.9 – 82.4 %. It is noteworthy that, in both cases, the highest reflectance values were obtained under ultra-fast cooling conditions.

**Figure S7** Calculation of cooling power density of 3D‑PVDF nanostructures on an aluminium substrate, with and without the AAO template, under different cooling methods.

**Figure S8** Emissivity in the 0.32 – 17 µm range for the two references: empty box (orange) and aluminium covered box (light grey), as well as the aluminium substrate placed beneath the 3D-PVDF nanostructures.

**Figure S9** Differential scanning calorimetry (DSC) curve of PVDF, with the crystallization temperature, *T_c_*, and melting temperatures, *T_m,α_* and *T_m,β_* marked.

**Figure S10** TGA thermograms of PVDF film, showing a decomposition temperature of 485.7 ºC.

**Cooling power density calculations**

$P_{cool}$ depends on the heat exchange between the cooler and its surroundings and can be calculated using the following expression:

$P_{cool}\left( T,T_{amb} \right)=P_{rad}\left( T \right)-P_{atm}\left( T_{amb} \right)-P_{sun}-P_{cc}\left( T,T_{amb} \right)$	(S1)

where $T$ is the cooler’s temperature and $T_{amb}$ the ambient temperature.

$P_{rad}\left( T \right)$ is the radiative power density:

$P_{rad}\left( T \right)=\iint_{0}^{\infty} I_{BB}\left( T,\lambda\right)\varepsilon\left( \lambda,\theta\right)d\lambda cos\theta d\Omega$	(S2)

where $I_{BB}\left( T,\lambda\right)$ is the spectral radiance density of a blackbody, $\varepsilon\left( \lambda,\theta\right)$ is the cooler’s emissivity, $\lambda$ is the wavelength, $\theta$ is the polar angle and $\int d\Omega$ is the integral over a hemisphere. Additionally, $I_{BB}\left( T,\lambda\right)$ is given by the following expression:

$I_{BB}\left( T,\lambda\right)=\frac{2hc^{2}}{\lambda^{5}}\frac{1}{e^{{hc}/{\lambda k_{b}T}}-1}$	(S3)

where $h$ is Planck’s constant, $c$ is the speed of light, and $k_{b}$ is Boltzmann’s constant.

$P_{atm}\left( T_{amb} \right)$ is the power density absorbed from atmospheric thermal radiation:

$P_{atm}\left( T_{amb} \right)=\iint_{0}^{\infty} I_{BB}\left( T_{amb},\lambda\right)\varepsilon\left( \lambda,\theta\right)\varepsilon_{atm}\left( \lambda,\theta\right)d\lambda cos\theta d\Omega$	(S4)

where $\varepsilon_{atm}\left( \lambda,\theta\right)$ is the atmosphere’s emissivity, that can be calculated using the following expression:

$\epsilon_{atm}\left( \lambda,\theta\right)=1-t\left( \lambda\right)^{1/{cos\theta}}$	(S5)

where $t\left( \lambda\right)$ is the atmosphere’s transmittance in the zenith direction.

$P_{sun}$ is the absorbed power density from solar radiation

$P_{sun}=\int\epsilon\left( \lambda,\theta\right)I_{AM1.5}\left( \lambda\right)cos\left( \theta_{sun} \right)d\lambda$	(S6)

where $I_{AM1.5}$ is the standard AM1.5G spectrum and $\theta_{sun}$ is the incidence angle of the solar irradiance.

Lastly, $P_{cc}$ is the power density that includes non-radiative effects (conduction and convection):

$P_{cc}\left( T,T_{amb} \right)=h_{cc}\left( T_{amb}-T \right)$	(S7)

where $h_{cc}$ is the heat-transfer coefficient.

**REFERENCES**

1.	J.Mandal, "Hierarchically Porous Polymer Coatings for Highly Efficient Passive Daytime Radiative Cooling," *Science* 362, no. 6412 (2018): 315-319, <https://doi.org/DOI>: 10.1126/science.aat9513

2.	Y. Zhang, J. Sun, Y. Wang, Y. Wu, C. Huang, C. Zhang and T. Liu, "A Radiative-Cooling Hierarchical Aligned Porous Poly(vinylidene fluoride) Film by Freeze-Thaw-Promoted Nonsolvent-Induced Phase Separation," *Chinese Journal of Polymer Science* 42, no. 7 (2024): 976-983, <https://doi.org/10.1007/s10118-024-3128-2>

3.	Q. Yue, L. Zhang, C.-Y. He, B.-H. Liu, W.-M. Wang, Z.-W. Lu, G. Liu and X.-H. Gao, "Polymer Composites with Hierarchical Architecture and Dielectric Particles for Efficient Daytime Subambient Radiative Cooling," *Journal of Materials Chemistry A* 11, no. 6 (2023): 3126-3135, <https://doi.org/10.1039/d2ta07453b>

4.	J. Park, D. Chae, H. Lim, J. Ha, C. Park, H. Sung, H. Song and H. Lee, "Highly efficient and versatile daytime radiative cooler based on optimized polymer-ceramic composite fabricated via facile process," *Solar Energy Materials and Solar Cells* 269, (2024): 112763, <https://doi.org/10.1016/j.solmat.2024.112763>
